# Long-term outcomes of 170 brain arteriovenous malformations treated by frameless image-guided robotic stereotactic radiosurgery: Ramathibodi hospital experience

**DOI:** 10.1097/MD.0000000000025752

**Published:** 2021-05-14

**Authors:** Pritsana Punyawai, Nicha Radomsutthikul, Mantana Dhanachai, Chai Kobkitsuksakul, Ake Hansasuta

**Affiliations:** a Chakri Naruebodindra Medical Institute, Faculty of Medicine Ramathibodi Hospital, Mahidol University, Samut Prakan; b Department of Surgery, Division of Neurosurgery, King Narai Hospital, Lopburi; c Department of Diagnostic and Therapeutic Radiology, Division of Radiation Oncology; d Department of Diagnostic and Therapeutic Radiology, Division of Interventional Neuroradiology; e Department of Surgery, Division of Neurosurgery, Faculty of Medicine Ramathibodi Hospital, Mahidol University, Bangkok, Thailand.

**Keywords:** arteriovenous, CyberKnife, frameless, malformation, radiosurgery, robotic, stereotactic

## Abstract

This study was conducted to report long-term outcomes of the frameless robotic stereotactic radiosurgery (SRS) for brain arteriovenous malformation (AVM) at Ramathibodi Hospital.

Retrospective data of patients with brain AVM (bAVM), who underwent CyberKnife SRS (CKSRS) at Ramathibodi Hospital from 2009 to 2014, were examined. Exclusion criteria were insufficient follow-up time (<36 months) or incomplete information. Patients’ demographics, clinical presentation, treatment parameters, and results were analyzed. Excellent outcome was defined as AVM obliteration without a new neurological deficit. Risk factors for achieving excellent outcome were assessed.

From a total of 277 CKSRS treatments for bAVM during the 6 years, 170 AVMs in 166 patients met the inclusion criteria. One hundred and thirty-nine cases (81.76%) presented with hemorrhages from ruptured bAVMs. Almost two-thirds underwent embolization before radiosurgery. With the median AVM volume of 4.17 mL, three-quarters of the cohort had single-fraction CKSRS, utilizing the median prescribed dose of 15 Gray (Gy). In the multisession group (25.29%), the median prescribed dose and the AVM volume were 27.5 Gy and 22.3 mL, respectively. An overall excellent outcome, at a median follow-up period of 72.45 months, was observed in 99 cases (58.24%). Seven AVMs (4.12%) ruptured after CKSRS but 1 patient suffered a new neurological deficit. Two patients (1.18%) were classified into the poor outcome category but there were no deaths. Negative factors for excellent outcome, by multivariate regression analysis, were the male sex and multisession SRS delivery, but not age, history of AVM rupture, previous embolization, or AVM volume.

Despite relatively larger bAVM and utilizing a lower prescribed radiation dose, the excellent outcome was within the reported range from previous literature. This study offers one of the longest follow-ups and the largest cohorts from the frameless image-guided robotic SRS community.

## 1 Introduction

Arteriovenous malformation (AVM) of the brain is a common cause of spontaneous intracranial hemorrhage in the young. Current treatment options for brain AVM (bAVM) are observation, surgical excision, endovascular embolization, and stereotactic radiosurgery (SRS).^[1,2]^ Numerous publications, from the GammaKnife (GK) and linear accelerator (LINAC) SRS series, demonstrated the efficacy and safety of radiotherapy for bAVMs.^[3–13]^ In contrast, data from CyberKnife (Accuray, Sunnyvale, CA) (CK) frameless image-guided robotic SRS centers were much less abundant. Colombo et al reported early results in 279 patients, treated with CK robotic SRS, with a median follow-up of 31 months. Subgroup analysis of the 102 cases, who had a follow-up duration of ≥36 months, showed an overall 71.5% rate of AVM obliteration.^[14]^ Apart from this report, other studies by Gupta et al, Ding et al, Wowra et al, Oermann et al, and Feutren et al comprised 9, 11, 20, 26, and 48 subjects, respectively.^[15–19]^ Thus, to furnish results from a greater number of patients with a longer follow-up period, a database of patients undergoing AVM treatment by CyberKnife SRS (CKSRS) at Ramathibodi Hospital was evaluated.

## 2 Materials and methods

### 2.1 Data collection

After approval by the Institutional Review Committee, a retrospective study of patients with bAVMs who underwent CKSRS from 2009 to 2014 was undertaken. Cases with incomplete data were excluded. Pre-SRS demographics of each patient such as age, sex, history of hemorrhage, previous AVM treatment (surgery, embolization, or radiotherapy), neurological status and clinical presentation were recorded. In those with unruptured AVMs, their initial symptom(s), such as seizure or incidental finding, were documented. Patients’ age, AVM volume, by milliliter, and location were taken into account for the calculation of modified radiosurgery-based score (mRBAS) as per the following equation: AVM score = (0.1 × volume [mL]) + (0.02 × age [year]) + (0.5 × location [0 or 1]). One point was assigned for deep locations (brainstem, basal ganglion, and thalamus), whereas 0 points were given for the non-deep areas.^[20]^ The mRBAS were arranged into ranges of scores, ≤1, 1.01 to 1.5, 1.51 to 2 and >2, for further examination. The CKSRS treatment parameters from the included AVM were retrieved.

### 2.2 Radiosurgery planning and treatment technique

A moldable plastic, custom-made, mask was individually fitted for each patient before obtaining the CKSRS protocol, with 1.2 mm cuts, and a contrast-enhanced computerized tomography (CT) scan. In addition, selected series of magnetic resonance imaging (MRI) consisting of thin-sliced (1–3 mm) gadolinium-enhanced T1, proton-density, and contrast-enhanced magnetic resonance angiography (MRA) were uploaded to the CK planning station. Subsequent integration of the MRI/MRA with the CT was done. The target (AVM nidus) as well as critical structures were delineated before treatment planning. For bAVMs with a diameter <30 mm, or volume <15 mL, a single-fraction SRS was employed. For larger targets, a multisession, in 5 daily deliveries, SRS regimen was utilized. An appropriate prescribed radiation dose, 15 to 20 Gray (Gy), was selected for the single-fraction SRS plan whereas 4 to 6 Gy/fraction for 5 consecutive fractions was applied for the multisession regimen. The prescribed dose was typically assigned to the 50% to 75% isodose line. A ray-tracing algorithm was exercised for dose calculation.

### 2.3 Outcome assessments

After the CKSRS treatment, appointments for clinical evaluation were scheduled at a 6-month interval. MRI and MRA scans at 24 or 36 months were typically obtained to determine complete obliteration of the AVM. At that time, those with apparent nidus or remaining flow, evident by early draining vein(s), were examined by annual neurologic tests, MRI, and MRA. In patients with nondetectable AVM nidus and no venous outflow by MRI/MRA, cerebral angiography would have been performed except in the event of patient refusal. For post-CKSRS assessment of outcomes, the included cases must have had at least 36 months of follow-up duration. The outcome of each patient, at his/her latest follow-up, was determined by the classification described by Pollock and Flickinger as follows: excellent, good, fair, unchanged and poor outcome, and death. Complete obliteration of the AVM without a new neurological deficit was defined as excellent outcome. Patients with AVM obliteration were classified into good outcome if they had minor deficit, and into fair outcome if they suffered major deficit that resulted in a decline of their functional status. If the AVM was not obliterated, the unchanged outcome was given to those without a new deficit while poor outcomes were patients who sustained a new deficit. Death was the last category of outcomes if it was believed to be directly related to the AVM or the SRS treatment.^[21]^ The percentage of the aforementioned outcomes were stratified into groups based on the ranges of mRBAS,^[20]^ as described earlier. Owing to the nature of this retrospective chart review, the clinical and radiographic outcomes, documented by multiple examiners and radiologists, were not blinded.

### 2.4 Data analysis

Patient, treatment, and outcome data would be presented as mean ± standard deviation (SD) or median (interquartile range [IQR]), where appropriate, for continuous variables and as percentage for categorical variables. To investigate patient and treatment factors influencing an excellent outcome, the data were compared using the Student *t* test or the Mann–Whitney *U* test for continuous variables, and the *χ*^2^ or Fisher exact test for categorical variables. Univariate and multivariate analyses were utilized to identify predictors for excellent outcomes by the logistic regression model with odds ratios and 95% confidence intervals (CI) computation. Kaplan–Meier survival graph of the proportion of AVM obliteration over time would be generated by linear regression analysis. All statistical tests were performed with Stata version 14 software (StataCorp, College Station, TX). Statistical significance was considered with a *P* value <.05.

## 3 Results

From 2009 to 2014, there were 277 bAVM patients who underwent CKSRS at our institute. The excluded subjects were 22 patients whose data could not be retrieved and another 89 cases with insufficient follow-up (<36 months). This resulted in a study cohort of 166 patients, harboring 170 AVMs. Two cases underwent CKSRS twice during the study period. Both of them had a substantial volume reduction in their AVMs but did not achieve complete obliteration. The other 2 subjects had 2 AVM nidi at different locations. A summary of the patients’ demographics is shown in Table 1. Most of the patients (81.76%) suffered AVM rupture with intracranial hemorrhage. One hundred and nine AVMs (64.12%) had endovascular occlusion before CKSRS. Approximately half of them underwent ≥2 sessions of embolization.

**Table 1:** Demographics of the 166 patients, 170 AVMs, who underwent CyberKnife SRS.

Demographics	No. (%)^∗^
Age, y median (IQR)	26.5 (17–39)
Sex	
Female	94 (55.63)
Male	72 (43.37)
Presentation	
Hemorrhage (ruptured AVM)	139 (81.76)
Asymptomatic	2 (1.18)
Seizure	29 (17.06)
Previous treatment	118 (69.41)
Previous embolization	109 (64.12)
1 Time	53 (48.62)
2 Times	30 (27.52)
3 Times	13 (11.93)
≥4 Times	13 (11.93)
Previous surgery	7 (4.12)
Previous SRS/radiotherapy	14 (8.24)

AVM = arteriovenous malformation, IQR = interquartile range, SRS = stereotactic radiosurgery, y = year.

∗ Number with percentage in brackets unless specified otherwise.

Table 2 summarizes the treatment characteristics of the reviewed cases. The majority (74.71%) of the bAVMs received single-fraction CKSRS with the median prescribed and maximum radiation doses of 15 (15–16) and 25.4 (22.9–27.6) Gy, respectively. The median AVM volume for single-fraction treatment was 4.17 (2.19–9.2) mL. Forty-three patients (25.29%) received a hypofractionated regimen, in 5 sessions. Their median prescribed and maximum doses were 27.5 (25–28) and 43.6 (38.2–45.3) Gy, respectively. The median AVM volume in this multisession cohort was 22.3 (13.39–37.86) mL.

**Table 2:** Stereotactic radiosurgery treatment characteristics of the AVM.

	Single fraction^∗^	5 Fractions^∗^	Overall^∗^
No. (%)	127 (74.71)	43 (25.29)	170 (100)
Treatment parameters			
AVM volume, mL	4.17 (2.19–9.2)	22.3 (13.39–37.86)	7.365 (2.53–12.71)
AVM location^†^:			
Deep, n (%)	28 (22.05)	9 (20.93)	37 (21.76)
Non-deep, n (%)	99 (77.95)	34 (79.07)	133 (78.24)
Prescribed dose, Gy	15 (15–16)	27.5 (25–28)	N/A
Maximum dose, Gy	25.4 (22.9–27.6)	43.6 (38.2–45.3)	N/A
Isodose line (%)	60.63 ± 6.11	64.19 ± 6.11	61.52 ± 6.32
AVM coverage (%)	94.82 ± 0.72	94.82 ± 0.64	94.82 ± 0.70
Conformity index	1.3 (1.21–1.48)	1.18 (1.14–1.22)	1.23 (1.17–1.37)

AVM = arteriovenous malformation, Gy = Gray, N/A = not applicable.

∗ The parameters are presented in median (interquartile range) or mean ± standard deviation unless specified otherwise.

† Based on modified radiosurgery-based scoring system (deep AVM locations = brainstem, basal ganglion, and thalamus).^[20]^

**Figure 1:**
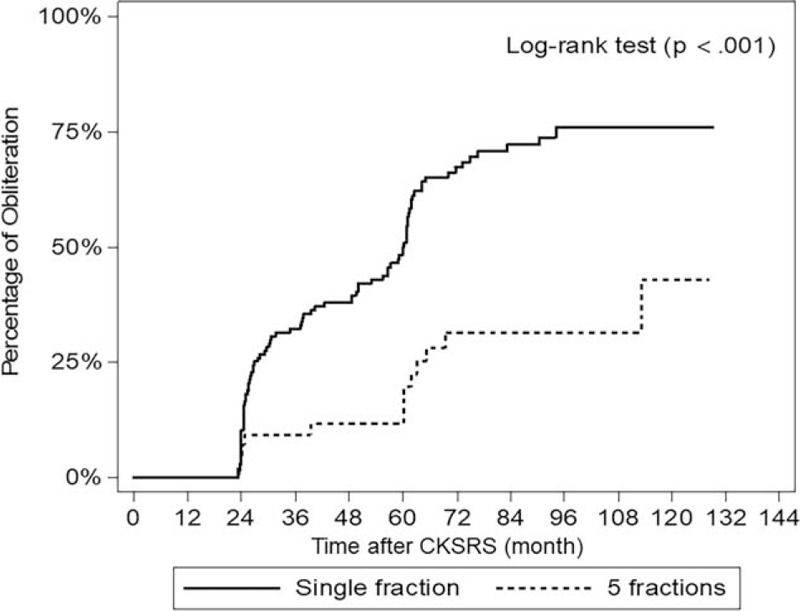
Kaplan-Meier curves demonstrating significantly different rates of arteriovenous malformation obliteration after single- vs 5-fraction CyberKnife stereotactic radiosurgery (CKSRS) treatments.

**Figure 2:**
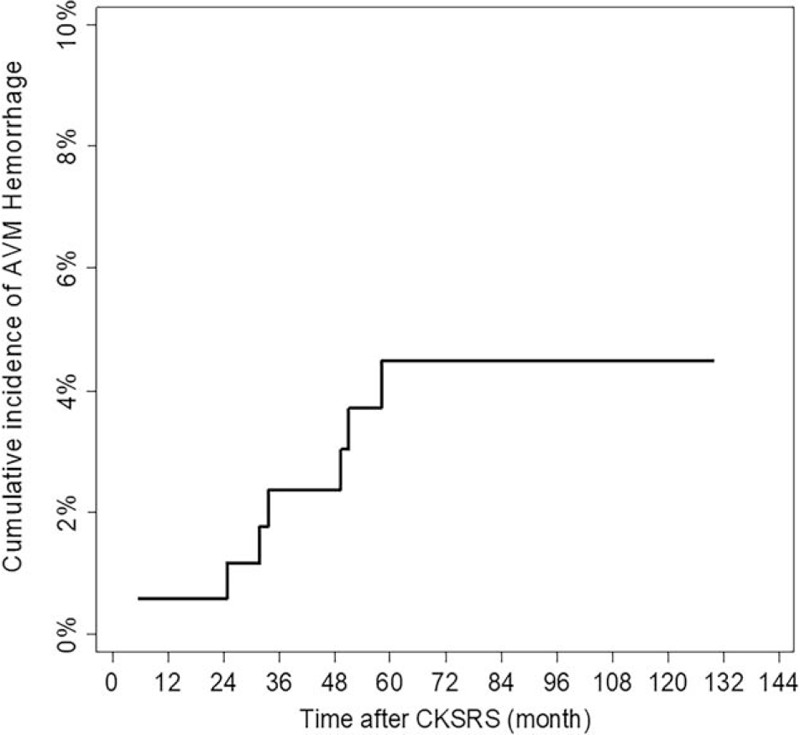
Cumulative incidence of arteriovenous malformation (AVM) bleeding after CyberKnife stereotactic radiosurgery (CKSRS) treatment.

With the median follow-up duration of 72.45 (60.7–91.8) months, 99 patients (58.24%) had complete AVM obliteration, confirmed by cerebral angiography in 72 (72.73%), and by MRI/MRA in 27 (27.27%) cases. The median time to obliteration was 39.4 (24.63–60.8) months (Table 3). The actuarial AVM obliteration rates from the single-fraction cohort, at 3, 5, 8, and 10 years, were 32.28%, 50.04%, 75.99%, and 75.99%, respectively. Patients with multisession CKSRS did not achieve the same level of success, evident by Log-rank test (Fig. 1), as the single-fraction treatment group (*P* < .001). Its actuarial post-SRS AVM obliteration rates were 9.3%, 11.75%, 31.6%, and 43%, at 3, 5, 8, and 10 years, respectively. No patient with AVM eradication suffered a new neurological deficit; hence, the rate of excellent outcome was maintained at 58.24% and none was classified into good or fair categories. Among the nonobliterated AVMs, 69 patients had no new deficit, resulting in the proportion of 40.59% for the unchanged outcome group. After CKSRS, 7 overall occurrences of AVM hemorrhage (4.12%), in 6 patients, were recorded. The events arose at as early as 6, but no later than 67 months after treatment (Fig. 2). Of the 6 patients, only 1 suffered a new neurological deficit with declined functional status. Apart from the hemorrhagic events, a 14-year-old girl developed glioblastoma multiforme (GBM), at the irradiated AVM region, 4 years after embolization and multisession CKSRS of her ruptured AVM. Surgical excision of the tumor was performed with subsequent chemotherapy and radiation. Although she did not have post-SRS hemorrhage or sustained a new neurological deficit, this patient and the aforementioned post-SRS hemorrhage victim were sorted into the same category, making the total number of 2 cases (1.18%) in the poor outcome group. Apart from the mentioned patients, the rest of the study cohort had no documented adverse radiation effect or death.

**Table 3:** Overall AVM obliteration and outcomes in the patients who underwent CKSRS.

	No.^∗^ (%)
Follow-up time, mo, median (IQR)	72.45 (60.7–91.8)
Overall AVM obliteration	99 (58.24)
AVM obliteration in single fraction CKSRS treatment group	86 (67.72)
AVM obliteration in 5-fraction CKSRS treatment group	13 (30.23)
Time to AVM obliteration, mo, median (IQR)	39.4 (24.6–60.8)
Post-CKSRS AVM hemorrhage	7 (4.12)
New neurological deficit	1 (0.59)
Glioblastoma multiforme	1 (0.59)
Outcomes	
Excellent	99 (58.24)
Good	0
Fair	0
Unchanged	69 (40.59)
Poor	2 (1.18)
Death	0

AVM = arteriovenous malformation, CKSRS = CyberKnife stereotactic radiosurgery, IQR = interquartile range, m = month.

∗ Number with percentage in brackets unless specified otherwise.

Table 4 details the patients’ and treatment's variables for excellent outcome. The significant factors were the male sex (*P* = .018), AVM volume (*P* = .021), multisession SRS (*P* < .001), isodose line (*P* = .018), and time to AVM obliteration (*P* < .001). In contrast to many publications, we did not find a history of AVM rupture (*P* = .672) or previous embolization (*P* = .632) to correlate with untoward results. Moreover, the age (P = .908) and deep location (*P* = .338) were not associated with excellent outcome, although the mRBAS appeared to be related (*P* = .013). Further evaluation by uni- and multivariate analyses for independent predictors of excellent outcome was performed. The AVM volume (95% confidence interval [CI] = 0.98–1.04, *P* = .488), mRBAS (95% CI = 0.27–2.31, *P* = .675), and isodose line (95% CI = 0.91–1.02, *P* = .272) were insignificant, by multivariate examination, whereas the male sex (95% CI = 0.25–0.99, *P* = .048) and multisession SRS (95% CI = 0.06–0.57, *P* = .003) were confirmed to negatively affect the outcome (Table 5).

**Table 4:** Analyses of variables for excellent outcome by SRS treatment of brain AVM.

	Total, n^∗^	Non-excellent outcome, n (%)^∗^	Excellent outcome, n (%)^∗^	*P*
AVMs	170	71 (41.76)	99 (58.24)	—
Patient factors				
Sex				**.018**
Female	97	33 (46.48)	64 (64.65)	
Male	73	38 (53.52)	35 (35.35)	
Age, y, mean ± SD		34.28 ± 14.42	34.03 ± 13.72	.908
Presentation:				
Hemorrhage (ruptured AVM)	139	57 (80.28)	82 (82.83)	.672
Seizure	29	12 (16.90)	17 (17.17)	.963
Asymptomatic	2	1 (1.41)	1 (1.01)	.999
Previous surgery				.701
No	163	69 (97.18)	94 (94.95)	
Yes	7	2 (2.82)	5 (5.05)	
Previous radiotherapy				.931
No	156	65 (91.55)	91 (91.92)	
Yes	14	6 (8.45)	8 (8.08)	
Previous embolization				.632
No	61	24 (33.80)	37 (37.37)	
Yes	109	47 (66.20)	62 (62.63)	
AVM volume, mL, median (IQR)	7.4 (2.5–12.7)	9.2 (3.1–16.3)	4.9 (2.2–11.5)	**.021**
AVM location:				.337
Deep (brainstem, basal ganglion, and thalamus)	37	18 (25.35)	19 (19.19)	
Non-deep	133	53 (74.65)	80 (80.81)	
mRBAS, median (IQR)	1.4 (0.9–2.0)	1.6 (1.1–2.3)	1.2 (0.8–1.9)	**.013**
mRBAS:				**.003**
mRBAS ≤1	47	15 (21.13)	32 (32.32)	
mRBAS 1.01–1.5	48	15 (21.13)	33 (33.33)	
mRBAS 1.51–2	32	22 (30.98)	10 (10.10)	
mRBAS >2	43	19 (26.76)	24 (24.24)	
Treatment factors				
SRS fractionation				**<.001**
Single fraction	127	41 (57.75)	86 (86.87)	
5 Fractions	43	30 (42.25)	13 (13.13)	
Single-fraction prescribed dose, Gy, median (IQR)	15 (15–16)	15 (15–16)	15 (15–16)	.997
Single-fraction maximum dose, Gy, median (IQR)	25.4 (22.9–27.6)	24.2 (22.4–27.3)	25.4 (23.3–27.7)	.254
5-Fraction prescribed dose, Gy, median (IQR)	27.5 (25–28)	26.7 (25–28)	27.5 (27–28)	.145
5-Fraction maximum dose, Gy, median (IQR)	43.6 (38.2–44.8)	43.6 (37.9–45.3)	43.1 (42.2–44.8)	.853
Isodose line, % mean ± SD	61.52 ± 6.32	62.87 ± 6.76	60.56 ± 5.83	**.018**
AVM coverage, %, mean ± SD	94.82 ± 0.70	94.88 ± 0.85	94.77 ± 0.57	.347
Conformity index, median (IQR)	1.2 (1.2, 1.4)	1.2 (1.2, 1.3)	1.2 (1.2, 1.4)	.320
Time to AVM obliteration, mo, median (IQR)	60.4 (30.5–75.2)	76.0 (60.8–106.1)	39.4 (24.6–60.8)	**<.001**

AVM = arteriovenous malformation, Gy = Gray, IQR = interquartile range, m = month, mRBAS = modified radiosurgery-based AVM score^[20]^, N/A = not applicable, SD = standard deviation, SRS = stereotactic radiosurgery, y = year.

∗ Number with percentage in brackets unless specified otherwise.

**Table 5:** Regression analyses of the predictors for excellent outcome by SRS treatment of brain AVM.

	Univariate analysis		Multivariate analysis	
	Odds ratio (95% CI)	*P*	Odds ratio (95% CI)	*P*
Patient factors				
Sex, male	0.474 (0.25–0.88)	.019	0.505 (0.25–0.99)	**.048**
Age	0.998 (0.97–1.02)	.908	—	
AVM volume	0.973 (0.95–0.99)	.023	1.010 (0.98–1.04)	.488
Deep AVM locations	0.699 (0.34–1.45)	.338	—	
mRBAS	0.770 (0.62–0.95)	.019	0.796 (0.27–2.31)	.675
Previous hemorrhage (ruptured AVM)	1.184 (0.54–2.59)	.672	—	
Previous embolization	0.855 (0.45–1.62)	.632	—	
Treatment factors				
Multisession SRS	0.206 (0.09–0.43)	<.001	0.185 (0.06–0.57)	**.003**
Isodose line	0.942 (0.89–0.99)	.020	0.969 (0.91–1.02)	.272

CI = confidence interval, mRBAS = modified radiosurgery-based AVM score,^[20]^ deep AVM locations = brainstem, basal ganglion, and thalamus, SRS = stereotactic radiosurgery.

## 4 Discussion

Stereotactic radiosurgery is an established treatment modality for bAVM.^[1,2]^ Ideal SRS ought to yield high rates of AVM obliteration with a trivial proportion of complications. Despite advances in imaging studies and radiation delivery techniques, AVM obliteration rates remained relatively unchanged. However, newer technologies appeared to have lowered the overall sequelae of radiotherapy.^[22]^ Thanks to the frameless immobilization, CyberKnife SRS permits the option of administering treatment, by either single- or multisession, for varying sizes of AVMs. However, unlike ample data by GK and LINAC series, the literature search, for full-text documents published in the English language, produced just <10 publications from CK series.^[14–19]^ Moreover, there was only 1 publication that had a median follow-up duration >60 months. Unfortunately, only 9 patients comprised this published data by Gupta et al.^[15]^ Our study, therefore, included both the large number of AVM subjects and the long follow-up time. The overall AVM obliteration rate in this study was in line with the previously reported range of 50% to 90%,^[3,4,8,11,12,23,24]^ albeit on the lower end of the spectrum, possibly due to the lesser-than-average prescribed dose (15 Gy) along with the larger target volume. The overall rate of post-SRS hemorrhage was also within the reported 1% to 5% range.^[2,7,14,17–19]^

Recognized negative factors, for AVM eradication by SRS, include patient factors, such as age, deep location, history of hemorrhage or large AVM volume, and treatment factors, that is, prescribed dose.^[7,8,25]^ This study concurred with previous publications with regard to the AVM volume and multisession SRS. It should reflect the substantial magnitude of targets that automatically mandated fractionation of radiotherapy rather than the poor selection of treatment options. On the other hand, the excellent outcome was not inversely affected by age, history of AVM rupture and deep location, as previous studies validating radiosurgery-based AVM scoring systems might suggest.^[26–28]^ Although most of the SRS series did not find sex to be associated with outcomes, this study, in a multivariate analysis, identified the male sex as an independent negative predictor. Frager et al published a similar observation^[29]^ and Bir et al found that female patients had a higher proportion of AVM obliteration.^[30]^ On the contrary, Yang et al found the male sex to be one of the protective factors against the post-SRS rebleeding^[31]^ and Liscak et al^[3]^ demonstrated that male patients achieved a higher percentage of AVM obliteration.

In addition to the above-mentioned negative factors, it is largely well-known that pre-SRS embolization hinders the probability of AVM obliteration.^[32,33]^ At Ramathibodi Comprehensive Neurovascular Center, there has always been a significant proportion of AVM patients whose pre-SRS embolization were necessary. In contrast, our statistical analysis contradicted those facts. Similar findings, of no untoward effect from prior embolization, were previously published by few centers.^[34,35]^ Oermann et al noted that the previously embolized AVMs had a significantly worse rate of obliteration after SRS. However, upon multivariate analysis, it failed to prove the case but, instead, the AVM architectural complexity was the actual negatively-affecting variable.^[36]^ We have not explored this particular matter in the present study.

By not including abstract-only information, an English language literature search for full-text, from the PubMed, Scopus, and Google Scholar databases, returned 4 CKSRS for bAVM series with at least 20 cases.^[14,17–19]^ Despite the largest number of subjects in their study, Colombo et al performed the assessments of outcomes from 102 patients who had at least 36 months of follow-up.^[14]^ Therefore, with 170 AVMs and the median follow-up duration of 72.45 months, our study represents the largest cohort demonstrating long-term results among the published series of frameless image-guided robotic SRS for bAVM (Table 6). Although the overall AVM obliteration from our study, compared with others, appeared to be relatively low, there are several conceivable explanations for it. First, the median AVM volume of 4.17 mL in the single-fraction group was the largest among the CKSRS series. In addition, this study comprised a higher proportion of larger AVMs than other CK series, with the median volume of 22.3 ml. Due to sizeable AVMs, the prescribed doses for single- and multisession CKSRS were relatively lower than in other studies. Considering this, lower than average obliteration rates were rather predictable. Nevertheless, the incidence of post-SRS AVM hemorrhage appeared to be within the reported range.^[14,17–19]^ Unfortunately, because different bodies of literature described various but not standardized outcomes, it was rather difficult to directly compare complication rates among the CKSRS series, other than the post-SRS AVM rebleeding. None detailed their results based on RBAS or mRBAS systems; hence, this study was the first, among CKSRS cohorts, to stratify results by standardized method. One patient with GBM was observed in the study. She was the second case who developed this malignancy after CKSRS for bAVM, after the first patient report from Xhumari et al.^[37]^

**Table 6:** CKSRS for brain AVM series with at least 20 cases including the present study.

	Colombo et al,^[14]^ 2009	Wowra et al,^[17]^ 2009	Oermann et al,^[18]^ 2014	Feutren et al,^[19]^ 2018	Present study
Parameters^∗^					
No. of patients treated with CKSRS	279	20	26	48	277
No. of patients with follow-up	267	20	26	48	255
No. of patients with follow-up >36 mo	102	NS	NS	33	166 Patients, 170 AVMs
Follow-up duration, mo	31	25	25	41	72.45
Patient characteristics					
Age, y	34	33.4	41	32	26.5
Patients with AVM rupture (hemorrhage), n (%)	45 (44.12)^†^	9 (45)	14 (54)	19 (39.58)	139 (81.76)
Proportion of patients with previous embolization (%)	50^†^	30	42.3	85.42	64.12
Overall AVM obliteration rate (%)	71.5^†^	67	57.69	68	58.24
RBAS (median)	1.41^†^	1.35	NS	1.24	1.4 (mRBAS)
<1, n (%)	17 (16.67)^†^	NS	NS	14 (29.2)	47 (27.65)
1.01–1.5, n (%)	37 (36.27)^†^	NS	NS	26 (54.2)	48 (28.24)
1.51–2, n (%)	30 (29.41)^†^	NS	NS	7 (14.5)	32 (18.82)
>2, n (%)	18 (17.65)^†^	NS	NS	1 (2.15)	43 (25.29)
Treatment parameters					
Proportion of patients treated with single-fraction CKSRS (%)	79.41^†^	100	100	100	74.71

					Single-fraction	5 Fractions
AVM volume, mL	1.95	1.8	1.62	2.6	4.17	22.3
Prescribed dose, Gy	18.7	22	19	NS	15	27.5
Maximum dose, Gy	25.5	30.3	NS	25	25.4	43.6
IDL (%)	NS	67	80	NS	60	64
CI	NS	NS	NS	NS	1.3	1.18

Outcome (%)					
Excellent	NS	NS	NS	NS	58.24
Good	NS	NS	NS	NS	0
Fair	NS	NS	NS	NS	0
Unchanged	NS	NS	NS	NS	40.59
Poor	NS	NS	NS	NS	1.18
Death	1	0	0	0	0
Remarks	1 Patient with new deficit	1 Patient with new deficit	3 Patients with motor deficit	2 Patients with grade 4 symptomatic ARE	1 Patient with GBM and 1 patient with new deficit
Overall post-CKSRS hemorrhage (%)	8 (3)^‡^	1 (5)	1 (3.8)	NS	7 (4.12)

ARE = adverse radiation effect, AVM = arteriovenous malformation, CKSRS = CyberKnife stereotactic radiosurgery, GBM = glioblastoma multiforme, m = month, mL = milliliter, mRBAS = modified RBAS,^[20]^ NS = not specified, RBAS = radiosurgery-based arteriovenous malformation score^[21]^.

∗ The parameters are presented in median unless specified otherwise.

† Outcome report from Colombo et al^[14]^ derived from 102 patients with follow-up >36 months.

‡ Post-CKSRS hemorrhage from Colombo et al^[14]^ was calculated from 8 incidences of 267 patients.

## 5 Study limitations

The presented study has some limitations. First, the “criterion standard” cerebral angiography to determine complete obliteration was not used in all cases. Due to the fact that some patients refused to take part in the post-SRS cerebral angiographic study, the outcome assessment is less than ideal because of the nonuniform post-treatment radiographic evaluation. Another constraint was the exclusion of 111 cases (39%) for lack of data or insufficient follow-up duration. It could have affected the overall obliteration rate or the incidence of complications as shown by Heffez et al.^[38]^ However, these limitations are common hindrances associated with retrospective reviews.

## 6 Conclusion

These results, with a considerable number of patients and extensive follow-up duration, confirmed the efficacy and safety of the frameless image-guided robotic stereotactic radiosurgery for brain AVM. Identified risk factors hindering achievement of excellent outcome were the male sex and multisession treatment.

## Acknowledgments

The authors thank Miss Suraida Aeesoa for her assistance in statistical analyses of the study and Dr. Nattaphong Rattanavirotkul for manuscript corrections.

## Author contributions

**Conceptualization:** Pritsana Punyawai, Mantana Dhanachai, Chai Kobkitsuksakul, Ake Hansasuta.

**Data curation:** Pritsana Punyawai, Nicha Radomsutthikul.

**Formal analysis:** Pritsana Punyawai, Ake Hansasuta.

**Investigation:** Pritsana Punyawai, Nicha Radomsutthikul.

**Methodology:** Pritsana Punyawai, Mantana Dhanachai, Ake Hansasuta.

**Project administration:** Ake Hansasuta.

**Supervision:** Mantana Dhanachai, Chai Kobkitsuksakul, Ake Hansasuta.

**Validation:** Pritsana Punyawai, Ake Hansasuta.

**Writing – original draft:** Pritsana Punyawai, Ake Hansasuta.

**Writing – review & editing:** Mantana Dhanachai, Ake Hansasuta.
